# Structure, Activity and Function of the PRMT2 Protein Arginine Methyltransferase

**DOI:** 10.3390/life11111263

**Published:** 2021-11-19

**Authors:** Vincent Cura, Jean Cavarelli

**Affiliations:** 1 Institut de Génétique et de Biologie Moléculaire et Cellulaire, 67404 Illkirch, France; cura@igbmc.fr; 2 Centre National de la Recherche Scientifique, UMR 7104, 67404 Illkirch, France; 3 Institut National de la Santé et de la Recherche Médicale, U1258, 67404 Illkirch, France; 4 Université de Strasbourg, 67000 Strasbourg, France

**Keywords:** protein arginine methylation, PRMT2, epigenetics, SH3, cancer

## Abstract

PRMT2 belongs to the protein arginine methyltransferase (PRMT) family, which catalyzes the arginine methylation of target proteins. As a type I enzyme, PRMT2 produces asymmetric dimethyl arginine and has been shown to have weak methyltransferase activity on histone substrates in vitro, suggesting that its authentic substrates have not yet been found. PRMT2 contains the canonical PRMT methylation core and a unique Src homology 3 domain. Studies have demonstrated its clear implication in many different cellular processes. PRMT2 acts as a coactivator of several nuclear hormone receptors and is known to interact with a multitude of splicing-related proteins. Furthermore, PRMT2 is aberrantly expressed in several cancer types, including breast cancer and glioblastoma. These reports highlight the crucial role played by PRMT2 and the need for a better characterization of its activity and cellular functions.

## 1. Introduction

Arginine methylation is a widespread posttranslational modification in eukaryotes catalyzed by protein arginine methyltransferases (PRMTs), a class of enzymes that transfers methyl groups from S-adenosyl-L-methionine (SAM) to guanidine nitrogen atoms in arginine residues of target proteins. 

Methylation makes arginine bulkier and more hydrophobic as well as reducing its H-bonding potential, thereby altering interactions with other proteins or nucleic acids [1,2]. Arginine methylation is involved in different cellular processes, including transcriptional regulation, RNA metabolism, DNA repair and signal transduction (see [2,3] for recent reviews). The nine PRMTs identified in mammals have been classified into three types. Type I PRMTs, including PRMT1, 2, 3, 4, 6 and 8, catalyze the formation of asymmetric dimethylarginine, while type II PRMTs (PRMT5 and PRMT9) produce symmetric dimethylarginine. PRMT7, the only type III PRMT, generates mono-methylarginine. 

This review is focused on PRMT2, one of the least functionally characterized PRMTs. The difficulty in detecting its importance in cellular processes was initially attributed to its low methyl transferase activity on classical PRMT substrates, namely, histone tails. However, various studies have since demonstrated the implication of PRMT2 in transcriptional regulation independently of its catalytic activity and, therefore, in cancer. Furthermore, recent results in the systematic analysis of PRMT interactomes shed new light on PRMT2 interactants and potential substrates [4]. The interaction of PRMT2 with RNA binding proteins and splicing factors is discussed.

## 2. Structural features

### 2.1. Sequence

**Figure 1:**
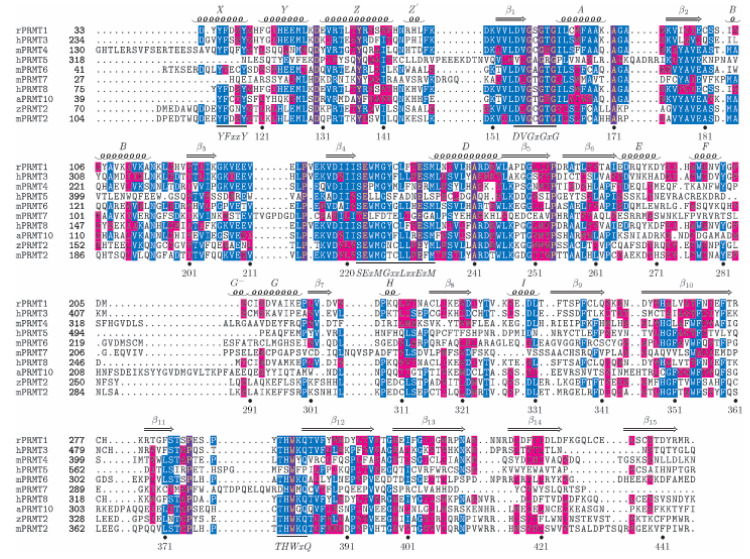
Structure-based sequence alignment of selected PRMTs. Ten PRMT sequences are aligned based on their crystal structures. The alignment is restricted to the catalytic core. The secondary structure of mPRMT2 is drawn above the alignment. The SAM-binding domain, the β-barrel domains and the dimerization arm are colored green, yellow and blue, respectively. The mPRMT2 residue numbering is shown below the sequences. The four signature sequences are localized, and their consensus is written below. Amino acids are shaded according to similarity to the consensus sequence. Amino acids highlighted are either invariant (violet) or similar (blue) as defined by the following grouping: F, Y and W; I, L, M and V; R and K; D and E; and G and A; S, T, N and Q. Abbreviations are as follows: m/Mus musculus, h/Homo sapiens, r/Rattus norvegicus, a/Arabidopsis thaliana and z/zebrafish (*D. rerio*). This figure, adapted from [10], was produced with the program TEXSHADE [11].

PRMT2 (or HRMT1L1) was first identified in the human genome through sequence homology with PRMT1 [5]. Phylogenetic analysis [6] and sequence comparisons have established that the PRMT2 methylation module is closely related to all type I PRMTs (35% to 39% sequence identity between PRMT1, PRMT3, PRMT6, PRMT8 and PRMT4 (CARM1) from mouse) (Figure 1). PRMT2 is present in all vertebrates, except in reptiles and birds, and has also been found in cnidaria, echinoderms and cephalochordates [7]. It is mainly localized in the nucleus, excluded from nucleolus, but is also found at low levels in the cytoplasm [8,9].

**Figure 2:**
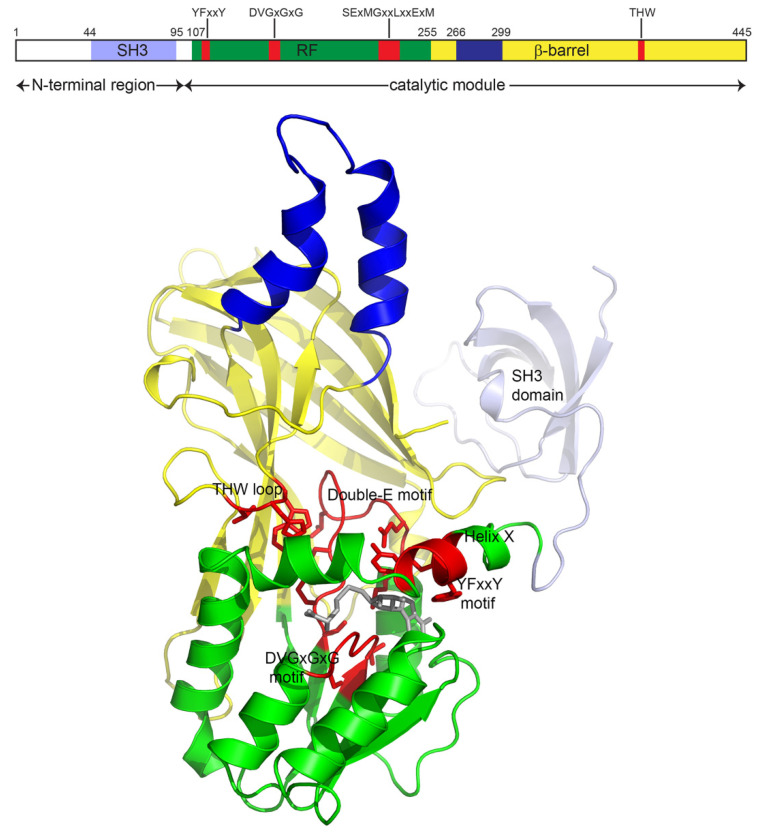
Full-length mPRMT2. Top: Scheme showing the modular organization of PRMT2. SH3 domain is indicated in light blue. The Rossmann fold is shown in green, the β barrel in yellow and the dimerization arm in blue. Motif YFxxY, motif DVGxGxG, double-E loop and motif THW are shown in red. Bottom: Three-dimensional model of full-length monomeric mouse PRMT2 generated with AlphaFold [12]. The modelized methylation module was replaced by the X-ray structure (PDB 5FUL) after superimposition. S-adenosyl-L-homocysteine (SAH) is displayed as a gray stick model. The 3D cartoon was generated with PyMol (http://www.pymol.org).

The PRMT2 sequence contains the canonical PRMT methylation core composed of two domains: An SAM-binding domain adopting a Rossmann fold followed by a β-barrel interrupted by a protruding helix–coil (Figure 2). PRMT2 exhibits all the conserved motifs involved in the SAM and peptide binding characteristics of PRMTs. However, these enzymes mainly differ in terms of the potential presence of additional domains. PRMT2 is characterized by an N-terminal extension containing a 50 residue Src homology 3 (SH3) domain located downstream of an unfolded N-terminal extremity that varies slightly in size, depending on the species.

**Figure 3:**
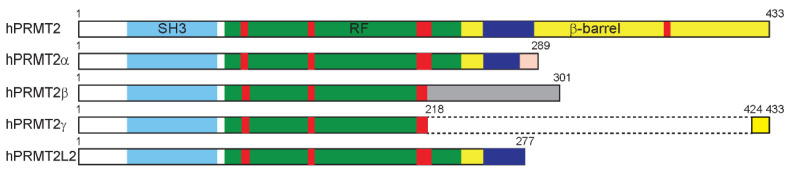
Human PRMT2 isoforms. PRMT2α is obtained after deletion of exons 8 to 10, including a modification of the 12 last residues due to a frameshift (in salmon). PRMT2β lacks exons 7 to 9, leading to a 301 amino acid sequence with a specific C-terminal sequence resulting from a frame-shift (in gray). The PRMT2γ isoform is produced by the removal of exons 7 to 10 corresponding to an in-frame deletion of 205 amino acids (indicated by dotted lines). PRMT2L2 being slightly smaller than PRMT2α results from alternative polyadenylation. Motif YFxxY, motif DVGxGxG, double-E loop and motif THW are represented in red. Dimerization helices are shown in blue.

Different isoforms resulting from alternative mRNA splicing have been found in several organisms. Nevertheless, according to sequence conservation, only one isoform is common to every species and is considered as the canonical form. In humans, in addition to the full-length PRMT2 expressed from a gene of eleven exons [5], six alternatively spliced PRMT2 isoforms have been detected (UniProtKB–P55345) and four of them (PRMT2L2, PRMT2α, β, and γ) have been isolated from breast cancer cells [13,14] (Figure 3). In all of these variants, the β barrel domain containing the dimerization helix–coil and the THW loop required for a fully active enzyme is missing. The sequences restricted to the SH3- and a major part of the Rossman-fold domain appear dramatically modified compared to the full-length PRMT2, leading to catalytically inactive proteins.

### 2.2. Structure

Several X-ray structures of the PRMT2 methylation core from two different organisms have been determined [10]. The structure of PRMT2 from *D. rerio* was solved with the co-factor product of the reaction S-adenosyl-L-homocysteine (SAH) (PDB: 5FUB) and sinefungin (PDB: 5G02), while the structure of *M. musculus* (mPRMT2) was obtained in complex with SAH (PDB: 5FUL) and three inhibitors (PDB: 5FWA, 5FWD and 5JMQ).

As expected, the monomeric structure of the PRMT2 catalytic module is very similar to that of all the known PRMT structures, especially of type I PRMTs. It consists of an SAM-binding domain (residues 107-254 and mPRMT2 numbering) adopting a Rossmann fold and a β-barrel domain composed of eight strands (residues 255–265 and residues 299–445) (Figure 2). The helical dimerization arm encompasses residues 266–298. The two domains are connected by the strictly conserved cis-proline 254 [15]. Unfortunately, the N-terminal module of mPRMT2 is missing in the electron density map despite being present in the crystal [10]. Similar wobbly domain behavior has previously been observed for the PH N-terminal domain of CARM1 [15]. However, a structure of the isolated SH3 domain of human PRMT2 was determined by NMR in 2005 (PDB 1X2P). It displays a classical SH3 fold containing 50 residues, which form five antiparallel β-strands folded into a barrel structure. SH3 domains are known to bind to target proteins through sequences containing proline and hydrophobic amino acids and are usually involved in protein–protein interactions [16,17].

### 2.3. Co-Factor Binding Site

**Figure 4:**
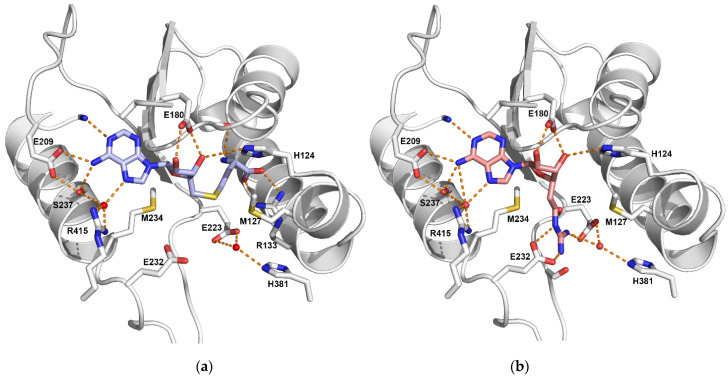
Interactions in the active site of mouse PRMT2 in complex with SAH (in blue) (PDB 5FUL) (**a**) and with Cp1 (in salmon) (PDB 5FWA) (**b**). The guanidinium group of Cp1 is held between the carboxylates of the double-E motif residues E223 and E232 by a set of H-bonds and salt bridges and mimics the arginine substrate guanidinium group. Water molecules are shown as red spheres, and hydrogen bonds are indicated by dotted lines. This figure was generated with PyMol (http://www.pymol.org).

The SAM binding pocket is formed by the motif sequence DVGCGTG (Figure 1). Hydrogen bonds involving the residue E209 carboxylate and the S237 Oγ maintain the SAM adenine amino group. N1 interacts with the V208 main chain carbonyl, and the E180 carboxylate forms two hydrogen bonds with the ribose hydroxyl oxygens. For the homocysteine moiety, the carboxylate group binds to R133, the amino group interacts with the C158 carbonyl, and M127 makes van der Waals contact with the S atom. Helix X, which harbors the conserved YFxxY motif, closes the SAM binding pocket (Figure 2 and Figure 4a).

### 2.4. Substrate Binding Pocket

The strictly conserved glutamate residues E223 and E232 of the double-E motif form a pair of salt bridges with the positively charged guanidinium group of the substrate arginine. These two glutamates are involved in the generally agreed PRMT catalytic mechanism by positioning the guanidinium group and modulating its nucleophilicity to favor methyl transfer [18,19].

PRMT2 structures obtained with SAM/arginine-like inhibitors indicate that the guanidinium group is positioned in the arginine pocket between the E223 and E232 carboxylates [10]. Helix X interacts via Y118 and Y114 with catalytic E232, together with the THW loop, allowing the formation of the substrate arginine pocket required for catalysis (Figure 2 and Figure 4).

### 2.5. Dimerization

**Figure 5:**
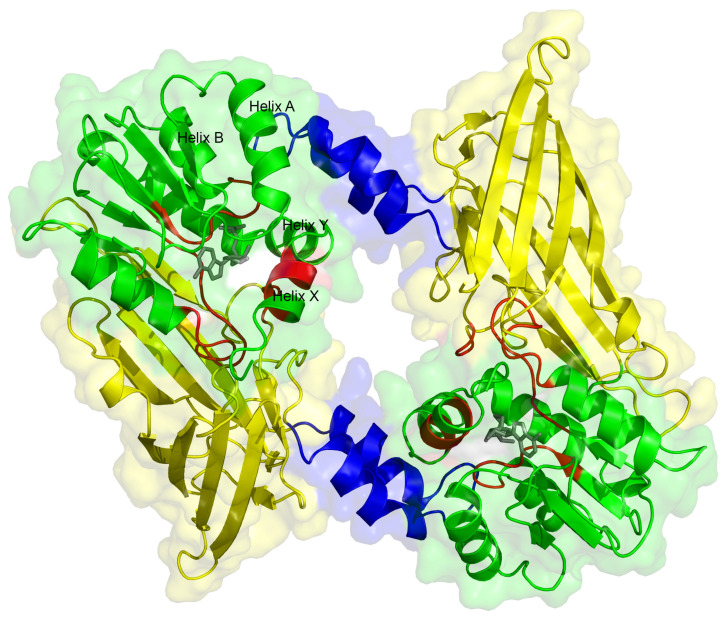
Crystal structure of dimeric mouse PRMT2 (PDB 5FUL). The N terminal part is not visible in the structure. The Rossmann fold domain is shown in green, the β barrel in yellow and the dimerization arm in blue. Helices A, B, X and Y interacting with dimerization helix–coil are indicated. SAH is displayed as a gray stick model, and SAM/SAH-binding motifs are in red. Structure cartoon was generated with PyMol (http://www.pymol.org).

Homodimerization is a feature conserved in all type I PRMTs and is essential for catalytic activity (see [3] for a recent review). In PRMT2, the dimer formation involves the dimerization arm from one monomer and helices Y, Z, A, and B from the other monomer, leading to the classical doughnut-shaped structure with a central hole, common to all type I PRMTs (Figure 5). Monomers are related to each other by a twofold rotational symmetry and are both able to bind the substrates. Small angle X-ray scattering (SAXS) experiments confirmed that PRMT2 behaves as a dimer in solution [10].

## 3. Activity

The first attempt to detect human PRMT2 (hPRMT2) activity proved unsuccessful. No activity could be detected using a recombinant hPRMT2, suggesting that the enzyme could be inactive [20]. Indeed, the in vitro methyltransferase activity of PRMT2 on histones is described as being weak compared to that of PRMT1, CARM1 and PRMT6 [21]. Initially, a small, but significant, activity was described on histone H4 [22]. Further experiments demonstrated that PRMT2 catalyzes asymmetric dimethylation of histone H3 arginine 8 in cells and that the presence of H3R8me2a at promoters is required to regulate target gene expression [23,24,25,26].

We detected a low methyltransferase activity signal using purified mPRMT2 and either H3 or H4 histone-tail peptides as substrates in vitro [10]. However, a stronger signal corresponding to PRMT2 automethylation could be revealed, suggesting that the enzyme is potentially fully active but that the optimal conditions, in terms of substrate or interacting partner, were not met. It is noteworthy that automethylation has been detected in several PRMTs [27,28,29,30]. A systematic analysis of protein methylation in mouse tissues revealed that R84 is methylated in mouse and human PRMT2 [31,32]. This arginine localizes in the SH3 domain and could, therefore, correspond to an automethylation site.

We discovered that an SAM-based compound, Cp1, reported as an inhibitor for PRMT1, PRMT6 and CARM [33], was also able to inhibit the activity of PRMT2 in vitro slightly more efficiently than SAH [10]. The IC_50_ values were 16.3 ± 3.8 µM for Cp1 and 18.3 ± 2.0 µM for SAH. However, thermal shift assays showed that binding of SAH increased PRMT2’s melting temperature (Tm) by 5.3 °C ± 1.2 °C with respect to the apo protein. The Tm shift reached 15 °C ± 1.9 °C with Cp1, indicating a stronger affinity of PRMT2 for Cp1 than for SAH. The X-ray structure revealed the key interactions occurring in the active site required for its recognition and specificity [10] (Figure 4b).

## 4. Interactions

### 4.1. Coactivation

It has been shown that PRMT2 acts as a coactivator of several nuclear hormone receptors. Using a yeast two-hybrid system, Qi and coworkers showed that PRMT2 interacts directly with estrogen receptor alpha (ERα) [34]. Three ERα regions, namely, AF-1, the DNA binding domain, and the hormone binding domain, were identified as interaction areas. The ER-interacting region on PRMT2, encompassing amino acids 133–275, is localized in the Rossmann fold domain. PRMT2 is able to enhance ER transcriptional activity. In another study, Meyer et al. [9] pointed out the interaction with ERα was strongly dependent on the cellular background, suggesting the involvement of differentially expressed coregulators.

The same authors identified PRMT2 as an AR-associated protein binding directly to the receptor via the C-terminal part (residues 271-433) of PRMT2 [9]. They demonstrated that PRMT2 acts as a strong coactivator of the androgen receptor (AR) in the presence of AR agonists. The coactivation function seems to depend on the methyltransferase activity of PRMT2. Furthermore, AR and PRMT2 colocalize and translocate from the cytoplasm into the nucleus when androgens are present.

PRMT2 promotes apoptosis by inhibiting NF-κB-dependent transcription [35]. The SAM-binding domain interacts with IκB-α by its ankyrin domain, which mediates the interaction with NF-κB. PRMT2 blocks nuclear export of IκB-α, causing increased levels of IκB-α in the nucleus and preventing NF-κB from binding DNA in mouse fibroblasts. The regulation role of PRMT2 on NF-κB was pointed out by Dalloneau and co-workers in the pulmonary inflammatory and airway distress syndrome induced by lipopolysaccharide (LPS) [36]. After LPS treatment, PRMT2 is downregulated in lungs and in macrophages, which allows the binding of NF-κB to the promoters of its target genes, such as cytokines IL-6 and TNF-α, leading to the inflammatory response.

In addition to its role as a transcriptional co-activator, PRMT2 has also been found to be involved in diverse cellular processes, such as energy homeostasis. The observation that PRMT2 null mice are leaner than wildtype animals, associated with perturbed energy metabolism, resistance to obesity and enhanced leptin sensitivity, suggested an involvement of PRMT2 in the regulation of feeding via a leptin-dependent pathway [37]. The authors showed that PRMT2 colocalizes with the transcription factor STAT3 in hypothalamic nuclei, where it binds and methylates STAT3 at the R31 residue. These results revealed that PRMT2 is a pivotal modulator of hypothalamic leptin–STAT3 signaling and energy homeostasis.

In 2015, Hussein and coworkers found that PRMT2 expression was reduced in diabetes-relevant high glucose conditions in macrophages. PRMT2 enhances ATP-binding cassette transporter A1 (ABCA1) expression induced by the liver X receptor (LXR) [38]. Thus, PRMT2 represents a glucose-sensitive factor that controls ABCA1-dependent cholesterol efflux and could provide a potential explanation behind the atherosclerosis development in diabetic patients. Although the mechanism is not known, this effect may be related to the discovery made by Li et al., who demonstrated that PRMT2 inhibits macrophage-derived foam cell formation [39].

PRMT2 interacts directly with PRMT1 to increase PRMT1 activity and influences the substrate specificity of the resulting complex both in vitro and in HeLa cells [40]. The binding requires the dimerization arm and catalytic activity of PRMT1. A study revealed that the SH3 domain regulates the interaction between PRMT1 and PRMT2 in a methylation-dependent manner. PRMT2 interacts with the retinoblastoma protein (RB) to regulate E2F transcriptional activity [41]. In contrast to other PRMTs, PRMT2 binds directly to RB through its SAM-binding domain, forming a ternary complex with E2F1. The authors of this study showed that PRMT2 repressed E2F1 transcriptional activity in an RB-dependent manner, delaying cell cycle progression from G1 to the S phase.

Blythe and coworkers showed that PRMT2 is directly recruited by β-catenin to target gene promoters during dorsal development in Xenopus, leading to histone H3 dimethylation on arginine 8 [23]. Associated with H3K4 trimethylation, H3R8me2 activates the Spindlin1-Wnt/β-catenin signaling pathway implicating the activity of PRMT2 in the expression of Wnt target genes [24].

Hou and coworkers showed that PRMT2 regulates the function of the actin nucleator Cobl by arginine methylation [42]. This posttranslational modification is crucial for proper Cobl association with G-actin. Both catalytic and SH3 domains are required for PRMT2–Cobl interaction and activity. The two methylated arginine residues are located in the second WH2 domain of Cobl, which is known to bind strongly to actin [43]. Thus, through Cobl methylation, PRMT2 plays a role in neuronal morphogenesis and dendritic arborization regulation in the central nervous system.

Additionally, PRMT2 expression was found to be selectively upregulated in alveolar epithelial cells of mouse lungs in response to chronic hypoxia. These results demonstrate that PRMT2 expression may be linked to asymmetric dimethylarginine metabolism [44].

### 4.2. Splicing

Protein arginine methylation is a posttranslational modification occurring on many proteins implicated in RNA processing [31]. In a systematic analysis of PRMT interactome, Wei and coworkers [4] found a significant enrichment for RNA binding domains in proteins interacting with PRMTs and revealed their importance in RNA splicing as well as in the assembly and function of ribosomes. These RNA-binding factors include heterogeneous nuclear ribonucleoproteins (hnRNPs) and serine/arginine-rich (SR) proteins that play a crucial role in pre-mRNA splicing. In these cases, arginine methylation constitutes a regulatory process controlling subcellular localization and protein–protein and RNA–protein interactions (see for reviews [45,46,47]). Thus, examples of the implication of PRMTs in RNA splicing have already been described: Sm proteins SmB/B0, SmD1, and SmD3 are methylated by PRMT5 [48,49]. RBM15, which regulates RNA export and splicing, is a substrate for PRMT1 [50]. CARM1 catalyzes the methylation of three splicing factors: SmB, U1-C and SF3B4/SAP49 [51]. PRMT9 forms a complex with splicing factors SF3B2/SAP145 and SF3B4/SAP49 and methylates SF3B2/SAP145 [52]. The methylation marks catalyzed by distinct PRMTs affect the subcellular localization of both serine/arginine-rich splicing factors 1 and 2 (SRSF1 and SRSF2, respectively) and occur between the two RRM domains in SRSF1 and in the RRM domain of SRSF2 [32,53]. They also impact the RNA binding functions of SRSF2.

Using a proteomic approach in HeLa cells, Vhuiyan and coworkers found associations between PRMT2 via the SH3 domain and different splicing-related proteins, some of which are also methylated by other PRMTs [54]. The list includes the Sm core snRNP protein SmB/B’; snRNP components; splicing regulators, such as hnRNPs; and other proteins involved in splicing, such as the heterogeneous nuclear ribonucleoprotein U-like 1 (HNRNPUL1), an hnRNP which represses basic transcription driven by several virus and cellular promoters, initially identified as an interactant by Kzhyshkowska et al [8]. The most characterized implication of hPRMT2 in splicing is the interaction with SAM68 (Src-associated in mitosis 68 kDa protein), a PRMT1 substrate that mediates the alternative splicing of the apoptosis regulator Bcl-X [54]. hPRMT2 promotes an increase in the BCL-X(L)/BCL-X(s) ratio in TNF-σ and LPS stimulated cells. This suggests an involvement of PRMT2 in regulating BCL-X alternative splicing in cells under inflammatory conditions and is consistent with the effect on the NF-κB pathway as previously described [35].

A few years ago, while purifying mouse PRMT2 from the insect expression host *Spodoptera frugiperda*, we found a 16 kDa contaminant co-eluting with mPRMT2 [10]. This polypeptide has been identified by mass spectrometry as repressor splicing factor 1 (RSF1). This insect-specific splicing repressor antagonizes serine and arginine-rich (SR) protein function [55] or coregulates alternative splicing with the other SR proteins in drosophila [56]. It contains an N-terminal domain folded into an RNA recognition motif (RRM) and a disordered arginine/glycine-rich C-terminal part. RSF1 is related to the serine/arginine-rich (SR) family of splicing regulators, in particular with the RRM domain of serine/arginine-rich splicing factors 7 and 3 (SRSF7 and SRSF3, respectively), which are involved in pre-mRNA splicing and mRNA export. Furthermore, six arginines methylated by PRMT2 were identified on RSF1, making it a usable substrate to detect PRMT2 enzymatic activity, as we showed with PRMT2 from mouse and *Danio rerio* [10]. However, it is still unclear whether PRMT2 releases the methylated RSF1 after the enzymatic reaction. In addition, the deletion of the SH3 domain leads to a sevenfold decrease in RSF1 methylation compared with the full-length enzyme, indicating that the SH3 domain could stabilize the interaction with RSF1. Thus, although RSF1 cannot be a natural substrate for PRMT2 due to the absence of this enzyme in insect cells, it could nevertheless shed light on the interaction between PRMT2 and a potential splicing regulator. 

## 5. Diseases

### 5.1. Breast Cancer

PRMT2 has been identified as a coactivator of several nuclear receptors, such as ERα and androgen receptors, which are involved in the development of hormone-dependent cancers [34]. The implication of PRMT2 in breast carcinogenesis has been described in several studies and remains complex. 

In addition to full-length human PRMT2, four alternatively spliced PRMT2 enzymatically inactive isoforms (PRMT2L2, PRMT2α, β and γ) have been identified [13,14] (Figure 3). Allof the PRMT2 isoforms showed increased expression in breast tumor compared to normal tissues and are all able to enhance ERα-mediated transactivation activity in the presence of estradiol. PRMT2L2 is predominantly localized in the cytoplasm, and PRMT2β exhibits an even distribution between the nucleus, including the nucleoli, and the cytoplasm, while full-length PRMT2, PRMT2α and γ are mainly present in the nucleus. This suggests that the alternatively spliced C-terminus would influence PRMT2 localization, while N-terminus extremity could control the transcriptional regulatory activity of PRMT2 isoforms. PRMT2 and PRMT2β expression suppresses the cell proliferation and colony formation of MCF7 cells, providing these isoforms with a tumor-suppressive role [57,58]. 

The loss of PRMT2 nuclear expression in breast cancer cells is linked to increased cyclin D1 expression via indirectly binding to the AP-1 site on the cyclin D1 promoter, thus promoting breast tumor cell proliferation. Inconsistently with these results, Ho et al. correlate PRMT2 depletion with decreased cyclin D1 expression [59].

The increased expression of the total amount of PRMT2 reported in breast cancer tissue could be explained by the high level of PRMT2 in the cytoplasm, since PRMT2 is clearly decreased in cell nuclei compared with normal breast tissue [58]. Thus, PRMT2 mRNA alternative splicing could be at least partially responsible for breast tumor development.

PRMT2 was able to reverse tamoxifen resistance in breast cancer cells generated by ER-α36, an estrogen receptor isoform lacking transcription activation functions AF-1 and AF-2 but still containing the DNA-binding domain and most of the hormone-binding domain [60]. This study revealed the interaction between PRMT2 and ER-α36 to suppress its non-genomic signaling pathways, PI3K/Akt and MAPK/ERK. Despite the confirmation of a direct association between PRMT2 and ER-α36, the PRMT2-mediated ER-α36 inhibition mechanism remains unknown.

While these studies all highlighted a critical role of PRMT2 expression in breast cancer, the mechanism remains widely unknown.

### 5.2. Other Pathologies

PRMT2 expression is upregulated in glioblastoma multiforme (GBM) [25] and in hepatocellular carcinoma (HCC) tissues and cells [26]. In both cases, PRMT2, through its catalytic product, H3R8me2a, is implicated in tumorigenesis. Hu et al. showed that PRMT2 is recruited to the Bcl-2 promoter and generates H3R8 dimethylation, which maintains Bcl-2 gene expression by inducing STAT3 accessibility, thereby promoting cell proliferation in HCC. 

Very recently, a decrease in PRMT2 expression in cardia gastric cancer tumors has been observed, which suggests a potential antitumor activity played by PRMT2 [61].

Zeng and coworkers revealed that PRMT2 provides protection against the proliferation of vascular smooth muscle cells and reduces the production of proinflammatory cytokines induced with angiotensin II [62]. These results show the ability of PRMT2 to reduce inflammation mediated by angiotensin II and suggest that it is as a potential target for cardiovascular diseases associated with vascular smooth muscle cell proliferation and inflammation.

## 6. Conclusions

PRMT2 is one of the least studied PRMTs, essentially because its methyl transferase activity is difficult to detect in vivo and no efficient substrate is available to determine enzymatic constants in vitro. RSF1 is, to date, the only interactant that can be used to reconstitute a complex with PRMT2 and that can be methylated in vitro. However, it is still unclear whether PRMT2 releases the methylated RSF1 after the enzymatic reaction, limiting its use in enzymology studies. It is therefore necessary to carry on investigations in order to identify an authentic substrate of PRMT2. On this point, techniques developed to analyze PRMT interactomes and methylomes succeeded in identifying interactants and substrates for different PRMTs and would certainly help in the discovery of substrates for PRMT2. This protein is known to interact with a multitude of splicing factors and splicing-related proteins, but there is no evidence of methylation by PRMT2, indicating possible functions that are independent of its catalytic activity. The role of the SH3 domain should also be clarified. This PRMT2-specific domain seems dispensable for PRMT2 coactivator function, but it has been demonstrated to be important for interactions with partner proteins. On this issue, isolation and structure determination of complexes would make a real breakthrough in the understanding of the SH3 domain’s function in PRMT2. Additionally, as a transcriptional coactivator of genes involved in oncogenesis, PRMT2 has been implicated in cancer pathogenesis and is, therefore, a potential target for cancer therapy. Thus, a better characterization of its physiological role in nuclear receptor signaling could encourage the development of therapeutic strategies.
